# Correction: Effects of aluminum chloride and coenzyme Q10 on the molecular structure of lipids and the morphology of the brain hippocampus cells

**DOI:** 10.1039/d1ra90168k

**Published:** 2021-11-08

**Authors:** Abdu Saeed, Safaa Y. Qusti, Rawan Hamdan Almarwani, Ebtihaj J. Jambi, Eida M. Alshammari, Naeem F. Qusty, Maha J. Balgoon

**Affiliations:** Department of Physics, Faculty of Science, King Abdulaziz University Jeddah 21589 Saudi Arabia Abdusaeed79@hotmail.com Abdusaeed@tu.edu.ye +96 6563190832; Department of Physics, Thamar University Thamar 87246 Yemen; Biochemistry Department, Faculty of Science, King Abdulaziz University Jeddah Saudi Arabia; King Fahd Medical Research Center Jeddah Saudi Arabia; Department of Chemistry, College of Sciences, University of Ha’il Ha’il 2440 Saudi Arabia; Medical Laboratories Department, Faculty of Applied Medical Sciences, Umm Al-Qura University Mecca Saudi Arabia

## Abstract

Correction for ‘Effects of aluminum chloride and coenzyme Q10 on the molecular structure of lipids and the morphology of the brain hippocampus cells’ by Abdu Saeed *et al.*, *RSC Adv.*, 2021, **11**, 29925–29933, DOI: 10.1039/D1RA03786B.

The authors regret that the name of one of the authors (Naeem F. Qusty) was shown incorrectly in the original article. In addition, the author contributions were incorrectly given. The corrected author list and contributions are as shown here.

## Author contributions

A. S. (Abdu Saeed): conceptualization, software, data curation, writing – review & editing. S. Y. Q. (Safaa Y. Qusti): conceptualization, supervision, writing – review & editing. R. H. A. (Rawan Hamdan Almarwani): methodology, investigation, experiment measurements, writing – original draft. E. J. J. (Ebtihaj J. Jambi): validation, writing – review & editing. E. M. A. (Eida M. Alshammari): validation, writing – review & editing. N. F. Q (Naeem F. Qusty): validation, writing – review & editing. M. J. B. (Maha J. Balgoon): conceptualization, supervision, writing – review & editing.

 

The Royal Society of Chemistry apologises for these errors and any consequent inconvenience to authors and readers.

## Supplementary Material

